# Microwave-Assisted Synthesis of Sulfur Quantum Dots for Detection of Alkaline Phosphatase Activity

**DOI:** 10.3390/nano12162787

**Published:** 2022-08-14

**Authors:** Fanghui Ma, Qing Zhou, Minghui Yang, Jianglin Zhang, Xiang Chen

**Affiliations:** 1 Hunan Provincial Key Laboratory of Micro & Nano Materials Interface Science, College of Chemistry and Chemical Engineering, Central South University, Changsha 410083, China; 2 State Key Lab of Powder Metallurgy, Central South University, Changsha 410083, China; 3 Department of Dermatology, Shenzhen People’s Hospital (The Second Clinical Medical College, Jinan University, The First Affiliated Hospital, Southern University of Science and Technology), Shenzhen 518020, China; 4 Department of Dermatology, Xiangya Hospital, Central South University, Changsha 410008, China

**Keywords:** sulfur quantum dots, nanomaterials, fluorescence sensing, alkaline phosphatase, ascorbic acid

## Abstract

Sulfur quantum dots (SQDs) are a kind of pure elemental quantum dots, which are considered as potential green nanomaterials because they do not contain heavy metal elements and are friendly to biology and environment. In this paper, SQDs with size around 2 nm were synthesized by a microwave-assisted method using sulfur powder as precursor. The SQDs had the highest emission under the excitation of 380 nm and emit blue fluorescence at 470 nm. In addition, the SQDs had good water solubility and stability. Based on the synthesized SQDs, a fluorescence assay for detection of alkaline phosphatase (ALP) was reported. The fluorescence of the SQDs was initially quenched by Cr (VI). In the presence of ALP, ALP-catalyzed hydrolysis of 2-phospho-L-ascorbic acid to generate ascorbic acid. The generated ascorbic acid can reduce Cr (VI) to Cr (III), thus the fluorescence intensity of SQDs was restored. The assay has good sensitivity and selectivity and was applied to the detection of ALP in serum samples. The interesting properties of SQDs can find a wide range of applications in different sensing and imaging areas.

## 1. Introduction

Fluorescent quantum dots (QDs) have been widely used in the fields of biosensing, cell imaging and biomedicine due to their unique size and superior optical properties [1,2,3,4,5,6,7,8,9]. However, heavy metal quantum dots such as CdS and CdTe are limited in practical applications due to their potential cytotoxicity and environmental hazard [10,11,12,13]. As a kind of pure elemental quantum dot, sulfur quantum dots (SQDs) have attracted much attention in recent years because of their low toxicity, good water solubility, stable optical properties and abundant raw materials [14,15,16,17,18,19]. So far, there have been reports on the application of SQDs in biosensing, cell imaging and antibacterial [20,21,22,23,24]. However, the complex and time-consuming synthesis process and the use of environmentally harmful substances make the synthesis of SQDs still a challenge, which limits its practical applications [25,26,27,28,29]. Therefore, it is necessary to develop green and time-saving methods to synthesize SQDs.

Alkaline phosphatase (ALP), as a membrane-bound enzyme widely exists in a variety of organisms, participating in the process of dephosphorylation in cells and hydrolyzing the phosphate groups of various substrates [30,31]. The normal level of ALP in adult blood is 40 to 190 U/L. Its abnormal expression is closely related to many diseases, such as bone diseases, liver dysfunction and various cancers. Serum ALP levels can be used as a reference for clinical diagnosis [32,33]. Therefore, it is of great significance to develop methods for effective detection of ALP. To date, surface-enhanced Raman scattering (SERS), electrochemical analysis, colorimetric detection, fluorescence analysis and other methods have been developed for the detection of ALP [34,35,36,37,38]. Among them, fluorescence detection technology has attracted significant attention because of its convenient operation, high throughput and high sensitivity.

Several fluorescent material-based chemical sensors have been developed for the detection and analysis of ALP [39]. For example, Li and co-workers designed a smartphone-based sensing strategy for ALP analysis using amino-functionalized copper (II)-based metal-organic frameworks (NH_2_-Cu-MOFs), which have both oxidase mimetic properties and fluorescence properties. The catalytic activity of NH_2_-Cu-MOFs was greatly inhibited due to the binding ability of Cu^2+^ with pyrophosphate (PPi). After the addition of ALP, the catalytic activity of NH_2_-Cu-MOFs was recovered due to the hydrolysis of PPi into orthophosphate by ALP, and then o-phenylenediamine was further catalyzed to form 2,3-diaminophenazine, which constituted a ratiometric fluorescent probe for the detection of ALP. The method has been successfully applied to the determination of ALP in serum samples. Zhang et al. constructed a novel near infrared ratiometric fluorescent probe (APT), which can achieve a rapid response to ALP (Within 10 min) [40]. After adding ALP, the fluorescence spectrum showed a shift (from 580 to 650 nm), and the near-infrared fluorescence emission (650 nm) made it more suitable for biological detection. The method has been successfully applied to the determination of ALP in serum and the detection and imaging of endogenous ALP in cells. In addition, nanocomposites have also been used to construct fluorescent sensors to detect ALP. Li’s group developed a simple hydrothermal method to construct “three-in-one” nanocomposites (Fef NCs) for the detection of ALP [41]. Fef NCs consist of three components, in which MnO_2_ nanosheets (NSs) are assembled on Fe_3_O_4_ nanoparticles (NPs), and then CeO_2_ NPs are modified. The nanometer material has various catalytic activity, and can realize label-free, ultrasensitive and selective detection of ALP by utilizing that characteristic. Duan et al. utilized WS_2_ quantum dots and MnO_2_ nanosheets to form a nanocomposite system to detect ALP [42]. MnO_2_ nanosheets can quench the blue fluorescence of tungsten disulfide quantum dots (WS_2_ QDs). However, in the presence of ALP and amifostine, their hydrolysis products triggered the decomposition of MnO_2_ nanosheets. This results in the restoration of fluorescence. Based on this discovery, the researchers successfully used the switch principle to detect ALP and used it in the analysis of actual samples. However, the above methods have limitations such as use of inorganic substances which limit their application and are harmful to the environment, and cumbersome synthesis or construction processes. Therefore, it is necessary to develop green, simple methods for efficient and sensitive detection of ALP.

**Scheme 1:**
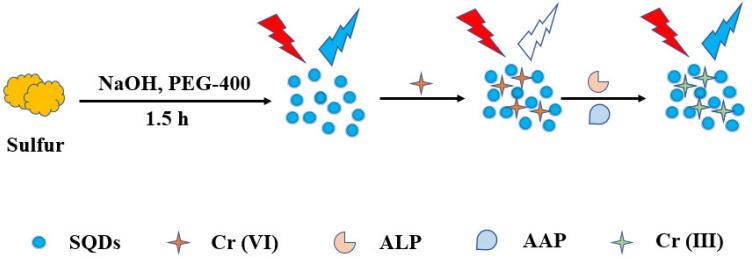
Synthesis of SQDs and schematic diagram for determination of ALP.

In this work, SQDs with stable optical properties were prepared by microwave-assisted heating using sublimed sulfur as a precursor and PEG-400 as a stabilizer. The synthesized SQDs was utilized for detecting ALP through a fluorescence “off-on” mechanism, as shown in Scheme 1. As the emission band of SQDs and the absorption band of Cr (VI) are well matched, there is a strong internal filtering effect (IFE) between them, so the addition of Cr (VI) can well quench the fluorescence of SQDs. However, ascorbic acid (AA) can reduce Cr (VI) to Cr (III), so the addition of AA can restore the fluorescence of the quenched SQDs. In addition, ALP can hydrolyze 2-phospho-L-ascorbic acid (AAP) to AA and phosphate ions; therefore, the activity of ALP can be detected via the recovery of the fluorescence of SQDs. Based on the above principle, the relationship between the fluorescence characteristics of sulfur quantum dots, Cr (VI), AA as well as ALP was explored, and the performance of SQDs for detection of ALP was studied in detail. The microwave synthesis of SQDs greatly shortens the synthesis time of SQDs and simplifies the operation steps, which have great reference significance for the exploration of the synthesis of SQDs. At the same time, the obtained SQDs are successfully used in the detection of ALP, which broadens the application of SQDs in biosensors.

## 2. Experimental Section

### 2.1. Materials and Apparatus

Potassium dichromate, sodium hydroxide, polyethylene glycol and sulfur powder were purchased from Aladdin (Shanghai, China). ALP and AA were obtained from Sigma-Aldrich (Shanghai, China). All reagents are of analytical grade and do not require further purification. The solutions used in the experiment were prepared by ultrapure water.

Transmission electron microscopy (TEM) images of the SQDs were obtained under a FEI Titan G2 60-300 microscope. Fluorescence measurements were performed on an F-7000 fluorescence spectrophotometer (Hitachi, Tokyo, Japan). UV-visible absorption spectra were recorded on a UV-2450 spectrophotometer (Hitachi, Tokyo, Japan).

### 2.2. Synthesis of SQDs

The synthesis of SQDs was based on previously reported methods with minor modifications [43,44,45]. Briefly, 1 g NaOH, 0.175 g sulfur powder, and 1.5 mL PEG-400 were mixed under stirring at 50 °C until the solution was clear. Then, the clear solution was placed in a microwave oven and reacted at 200 W for 1.5 h. Next, the obtained solution was centrifuged at 6000 rpm for 15 min, and the supernatant was centrifuged for two more times. Finally, the obtained supernatant was the SQDs solution, which was placed at 4 °C before use.

### 2.3. Determination of ALP

Initially, 20 µL of SQDs solution was mixed with various concentrations of aqueous K_2_Cr_2_O_7_, then a PBS buffer (pH 7.4, 0.1 M) was added until the volume of the mixture reached 200 µL. The fluorescence spectrum of the solution was then measured. The excitation wavelength was maintained at 380 nm throughout the detection.

For the detection of ALP, 40 µL of solution containing different concentrations of ALP were mixed with 40 µL of 30 mM AAP. After mixing, the solution was incubated for 30 min in a water bath at 37 °C, then 10 µL of 10 mM K_2_Cr_2_O_7_ was added. After incubating for another 10 min, 20 µL of SQDs was added into the mixture. Fluorescence spectra of the solution were then collected.

## 3. Results and Discussion

### 3.1. Preparation and Characterization of the SQDs

**Figure 1:**
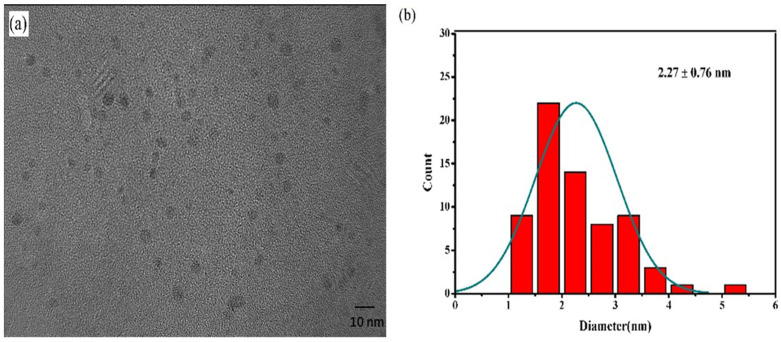
Morphological characterizations of the SQDs: (**a**) TEM images; (**b**) size distribution histogram.

As shown in Scheme 1, using sulfur powder as a precursor, and PEG-400 as stabilizer, SQDs were synthesized by microwave-assisted heating. The whole synthesis process is easy to operate, the raw materials used being basically non-toxic to the environment, and the synthesis time was shortened to 90 min. In order to understand the size and morphology of SQDs, the morphology of SQDs was imaged by transmission electron microscope (TEM). As shown in Figure 1a, SQDs can be well dispersed into water. The particles display spherical shape, and the particle size is 2.27 ± 0.76 nm, which is similar to the fluorescent SQDs previously reported.

The excitation and emission positions and fluorescence intensity of SQDs can be obtained by fluorescence tests. Fluorescence properties of the synthesized SQDs were studied (Figure S1). When excited under different wavelength, with the increase of wavelength from 320 nm to 380 nm, the emission intensity of SQDs increases with the increase of excitation wavelength. By further increasing the wavelength from 380 nm to 420 nm, the emission intensity decreases with the increase of excitation wavelength. In addition, the fluorescence emission peak is redshifted with the increase of excitation wavelength. Therefore, 380 nm was used as the optimal excitation wavelength in subsequent experiments.

### 3.2. Feasibility of the Assay for ALP Analysis

**Figure 2:**
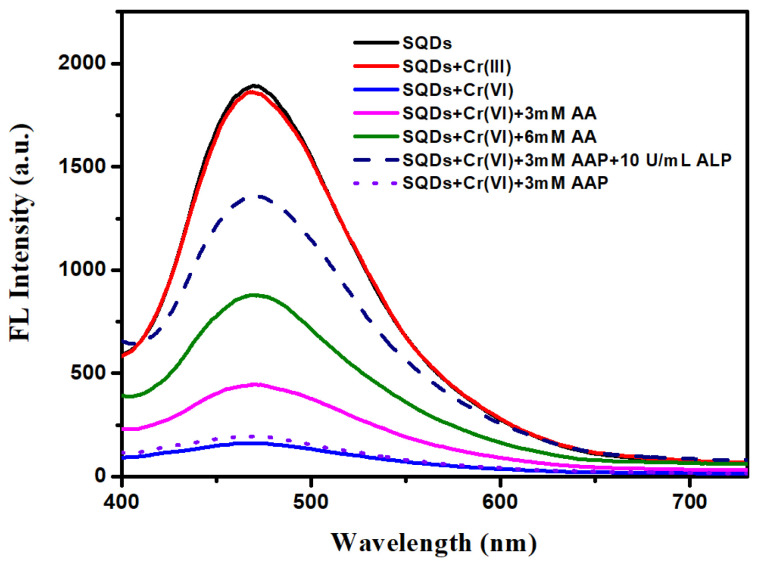
Feasibility analysis of the assay for ALP detection.

The feasibility of the assay for ALP analysis was tested. As described in Figure 2, SQDs has a strong fluorescence emission peak at about 470 nm. However, the fluorescence of the SQDs is quenched after adding a certain concentration of K_2_Cr_2_O_7_, and can then recovered with further addition of AA. The recovery of fluorescence intensity is in accordance with the amount of AA added; this is because AA can reduce Cr (VI) to Cr (III). The fluorescence intensity of SQDs, mixed with a certain amount of chromium chloride solution, has no obvious difference with that of the single SQDs solution, which indicates that Cr (III) generated due to the reduction of K_2_Cr_2_O_7_ by AA has no quenching effect on the fluorescence signal of the SQDs. Meanwhile, after the K_2_Cr_2_O_7_ is added into the SQDs, the fluorescence signal is not changed by following addition of AAP, indicating AAP can not react with K_2_Cr_2_O_7_ and AAP alone will not affect the fluorescence intensity of the system. However, in the presence of ALP, the fluorescence signal was restored, which verified that ALP can catalyze the hydrolysis of AAP to generate AA, thus achieving the same effect as adding AA. Based on the above experimental results, it can be seen that fluorescence sensing of ALP activity can be achieved based on SQDs.

**Figure 3:**
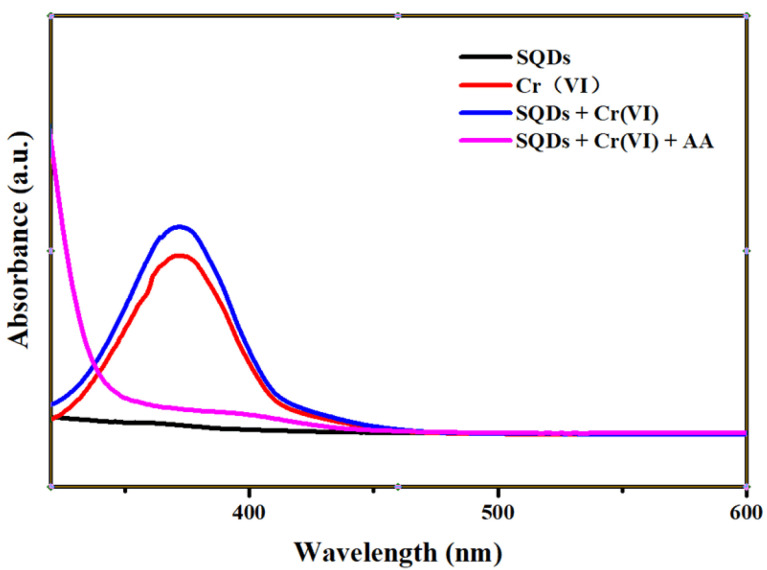
UV-vis absorption spectra of different solutions.

UV-vis absorption spectra further verified the above experimental results (Figure 3). Cr (VI) has a strong absorption at 380 nm. In contrast, the absorption of SQDs is weak. The competion of Cr (VI) ion with SQDs for the absorption of 380 nm light resulted in the quenching of the fluorescence of SQDs. Adding SQDs to Cr (VI), the absorption at 380 nm is slightly enhanced compared to Cr (VI) alone which is due to the absorption of SQDs at 380 nm. After the addition of AA, the absorption at this point almost disappeared because AA reduced Cr (VI) to Cr (III). These results are consistent with the fluorescence data.

### 3.3. Detection of Cr (VI)

**Figure 4:**
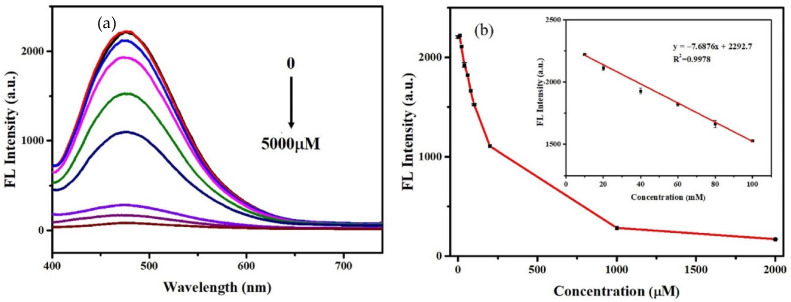
(**a**) Emission spectra of SQDs after adding different concentrations of Cr (VI) solution from 0 to 5 mM; (**b**) Fluorescence value of the SQDs in response to different concentrations of Cr (VI) in the range of 0 to 2 mM. Inset: the calibration curve to Cr (VI) in the linear range of 10–100 µM.

The quenching of the fluorescence of SQDs by Cr (VI) is due to fact that the excitation spectrum of SQDs overlaps well with the absorption band of Cr (VI). Therefore, a strong IFE occurred between SQDs and Cr (VI), because Cr (VI) can shield the excitation light of SQDs. As shown in Figure 4a, the fluorescence intensity of SQDs decreased gradually with the increase of the amount of Cr (VI) added. When the Cr (VI) concentration reaches 5 mM, the fluorescence of SQDs is almost completely quenched. The fluorescence intensity of SQDs was linearly correlated with Cr (VI) concentration in the range of 10–100 µM and has a good linear correlation coefficient (*R*^2^ = 0.997) (Figure 4b).

**Figure 5:**
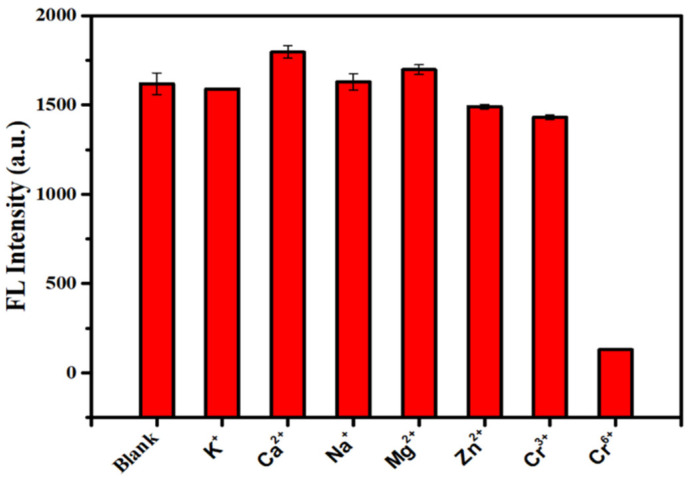
Response of SQDs to different ions.

The selectivity of the synthesized SQDs for Cr (VI) was evaluated. Under the same experimental conditions, 2 mM Cr (VI) and 6 mM, other interfering ions, were added into SQDs and the fluorescence intensity was measured under 380 nm excitation. As described in Figure 5, when 2 mM Cr (VI) was added to the SQDs, the fluorescence intensity of the SQDs was reduced by more than 90%. However, even when 6 mM of other common metal ions were added, the fluorescence intensity of the SQDs was not affected significantly. These data show that the SQDs have good selectivity for Cr (VI).

### 3.4. Detection of ALP

**Figure 6:**
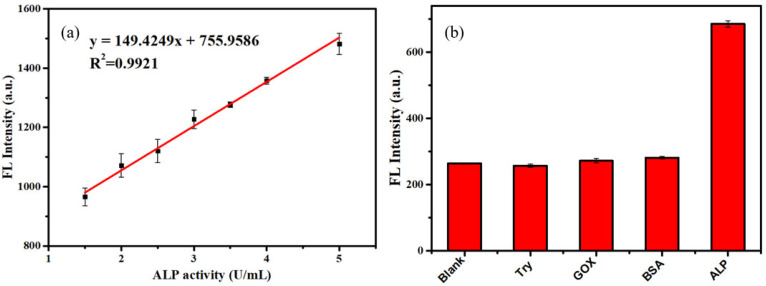
(**a**) Fluorescence response of the assay to ALP of different activities. Inset: linear response of SQDs to ALP in the activity range of 1.5–5.0 U/mL; (**b**) Selectivity of the assay to ALP.

The absorption peak of Cr (VI) at about 380 nm disappears after the addition of AA, which indicates that AA can reduce Cr (VI) to a low-valent Cr species, thereby eliminating the IFE between Cr (VI) and SQDs and restoring the fluorescence of SQDs. ALP can hydrolyze AAP to generate AA, which can also recover the fluorescence of SQDs-Cr (VI) system. Hence, the principle can be applied for detecting ALP. The selectivity of the system to AA was investigated. Other potential reductants including glutathione (GSH), cysteine (Cys), glucose and some common ions had little effect on the fluorescence of SQDs-Cr (VI) system, indicating that SQDs-Cr (VI) system had good selectivity for AA (Figure S2). As described in Figure 6a, the fluorescence intensity of SQDs increases with the increase of ALP activity. When the ALP activity is more than 10 U/mL, the fluorescence intensity of the system tends to be stable, and the fluorescence strength of SQDs was almost fully recovered. The fluorescence intensity of SQDs had a good linear relationship with ALP activity in the range of 1.5–5.0 U/mL, and the linear correlation coefficient R^2^ was 0.992. Based on a signal–noise ratio of 3, the calculated LOD was 0.13 U/mL. Table 1 summarizes previously reported strategies for detecting ALP using different probes, indicating that the methods are comparable to those reported in other studies.

Then, the selectivity of the assay for ALP was analyzed. Responses of the assay to 2 U/mL of alkaline phosphatase (ALP) and other enzymes with an activity of 5 U/mL were tested. As described in Figure 6b, when ALP is added, the fluorescence change is obvious, while the responses of the assay to other enzymes are negligible, similar to blanks. These data show that the assay has good selectivity to ALP sensing. In addition, the assay is used for the recovery testing of ALP in serum. With the serum signal as the background, ALP with the activity of 1, 2, 2.5 and 3.5 U/mL were added into the serum sample, and then the level of ALP was measured. The results are shown in Table 2. The recoveries for ALP in the serum were in the range of 102.01~119.61%, with the relative standard deviations (RSD) less than 5%. Therefore, the assay has potential application for clinical detection of ALP.

**Table 1:** Comparison employing fluorescent probes to detect ALP.

Probe	Linear Range/U·mL^−1^	LOD/U·mL^−1^	Ref.
Luminol– Tb–GMP CPNPs	0.00005–0.1	0.00002	[46]
Hydrogelator	0−2.8	0.06	[31]
NIR-Phos-1,NIR-Phos-2	0–1.0	10^−5^–10^−3^	[47]
5-bromo-4-chloro-3-indolyl phosphate	10–1000	0.87	[48]
CuNPs	0.1–40	0.05	[49]
SQDs	1.5–5.0	0.13	This work

## 4. Conclusions

In this work, by using sulfur powder as a precursor and polyethylene glycol as a stabilizer, SQDs were prepared successfully through a microwave-assisted method. The synthesis time of the SQDs was greatly shortened to only 90 min. The synthesized SQDs had good water solubility and good fluorescence stability. A sensitive and selective fluorescence assay based on IFE for detection of ALP was developed by using synthesized SQDs. In the presence of Cr (VI), the fluorescence of SQDs was quenched, and then recovered by the addition of AAP and ALP. The SQDs as fluorescence probes can find a wide range of applications in different sensing areas.

## Figures and Tables

**Table 2:** Recovery analysis of ALP in human serum.

Samples	Added (U/mL)	Found (U/mL)	Recovery (%)	RSD (%)
	1	1.43 ± 0.02	-	0.301
human serum	2	2.63 ± 0.09	119.61	0.495
	2.5	3.05 ± 0.20	108.15	2.497
	3.5	3.05 ± 0.20	102.01	1.181

## Data Availability

Data is contained within the article.

## Supplementary Materials

The following supporting information can be downloaded at: https://www.mdpi.com/article/10.3390/nano12162787/s1, Figure S1: Fluorescence emission spectra of SQDs under different excitation wavelength, Figure S2: Selectivity of the SQDs-Cr (VI) system for AA.
