# Supplementary material for: Microwave-Assisted Synthesis of Sulfur Quantum Dots for Detection of Alkaline Phosphatase Activity
Source: Nanomaterials (Basel). 2022 Aug 14;12(16):2787. doi: 10.3390/nano12162787 (PMC9414924; doi:10.3390/nano12162787)
Supplement: Supplementary file 1 [file nanomaterials-12-02787-s001.zip › nanomaterials-1793277-supplementary.pdf]

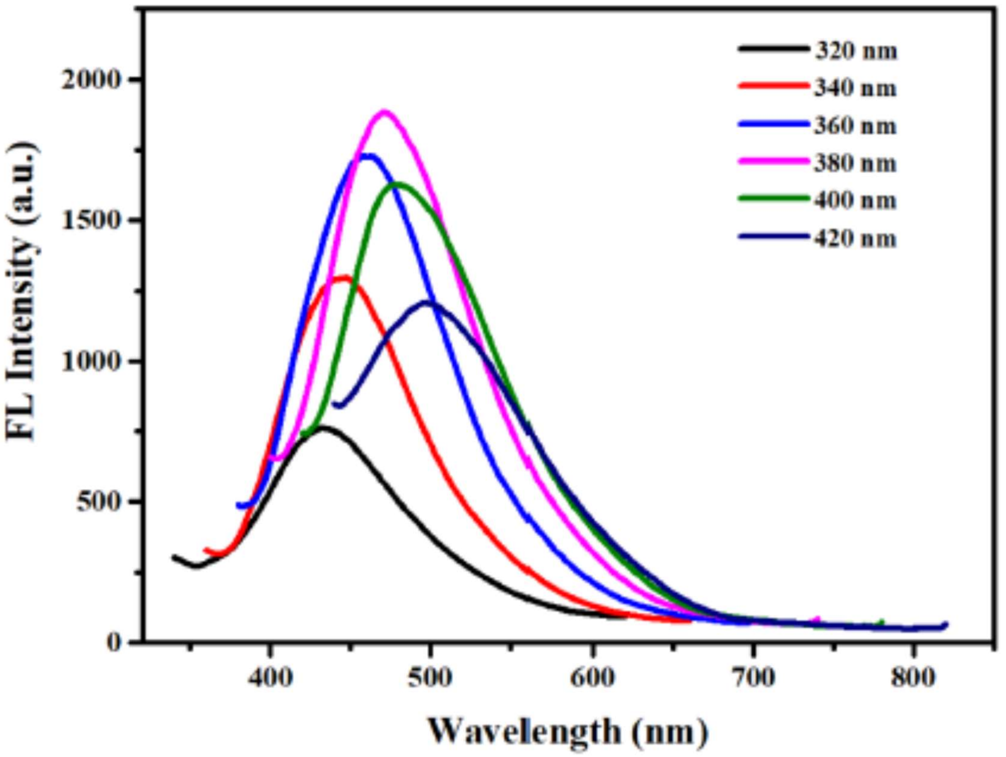

Figure S1. Fluorescence emission spectra of SQDs under different excitation wavelength.

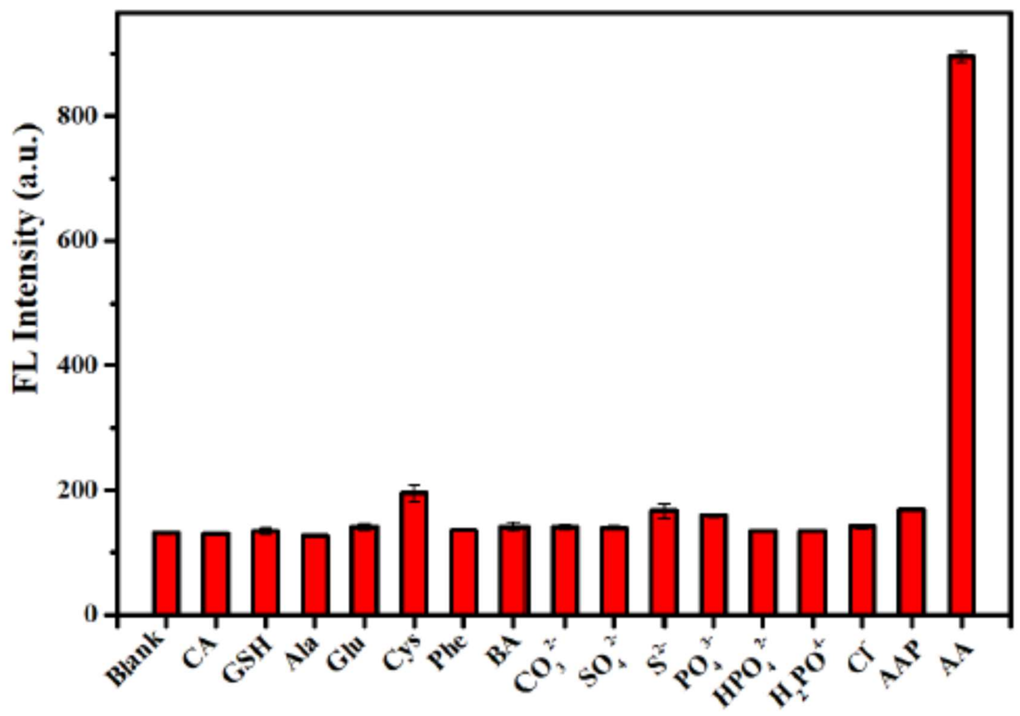

Figure S2. Selectivity of the SQDs-Cr (VI) system for AA.
